# Evidence From a Systematic Review of Non-alcoholic Fatty Liver Disease (NAFLD)/Metabolic Dysfunction-Associated Fatty Liver Disease (MASLD) Fueling Cardiovascular Risk

**DOI:** 10.7759/cureus.89355

**Published:** 2025-08-04

**Authors:** Nehal K Bhatt, Yashasvi Agarwal, Sanathanan Neelakantan Ramaswamy, Manvitha Bendagiri Matam, Samyuktha Harikrishnan, Shalvin Chand, Hetavi Bhatt, Iana Malasevskaia

**Affiliations:** 1 Internal Medicine, Pramukhswami Medical College, Karamsad, IND; 2 Internal Medicine, Sir H.N. Reliance Foundation Hospital and Research Centre, Mumbai, IND; 3 Internal Medicine, Jawaharlal Nehru Medical College, Belagavi, IND; 4 Internal Medicine, Government Erode Medical College and Hospital, Erode, IND; 5 Medicine, Gandhi Medical College, Secunderabad, IND; 6 Nephrology, St Helier Hospital, London, GBR; 7 Medicine, Gulf Medical University, Ajman, ARE; 8 Medicine, University of Fiji, Lautoka, FJI; 9 Research, California Institute of Behavioral Neurosciences & Psychology, Fairfield, USA; 10 Pharmacy, Parul University, Vadodara, IND; 11 Obstetrics and Gynecology, Private Clinic 'Yana Alexandr', Sana'a, YEM

**Keywords:** cardiovascular disease, fibrosis, metabolic dysfunction-associated steatotic liver disease, non-alcoholic fatty liver disease, systematic review

## Abstract

Non-alcoholic fatty liver disease (NAFLD), now termed metabolic dysfunction-associated steatotic liver disease (MASLD), is increasingly implicated as a key risk factor for cardiovascular disease (CVD). However, its independent contribution remains debated due to confounding metabolic factors and methodological heterogeneity. This review aims to synthesize evidence on the association between NAFLD/MASLD and cardiovascular outcomes, focusing on the influence of liver fibrosis and metabolic dysfunction.

Following Preferred Reporting Items for Systematic Reviews and Meta-Analyses (PRISMA) 2020 guidelines, we searched PubMed/MEDLINE, ScienceDirect, Europe PMC, and Cochrane Library (through May 2025) for observational studies and trials assessing NAFLD/MASLD and CVD outcomes in adults. NAFLD required imaging/biopsy or validated scores (fatty liver index (FLI) ≥30, Fibrosis-4 Index (FIB-4)); comparators were non-NAFLD controls. Outcomes included myocardial infarction, stroke, CVD mortality, and atherosclerosis. Two reviewers independently screened records, extracted data, and assessed risk of bias (Newcastle-Ottawa Scale (NOS)/Cochrane RoB 2).

The systematic search identified 21 eligible studies (18 cohort, two cross-sectional, and one RCT). Sample sizes ranged widely, from a few hundred in clinical cohorts to over 9.5 million in a national database study. Observational studies demonstrated good methodological quality (mean NOS score ≥7/9). NAFLD/MASLD showed a consistent dose-dependent association with cardiovascular risk, with effect sizes ranging from modest (adjusted HR=1.2) to substantial (HR=8.0). Risk stratification revealed (i) advanced fibrosis (FIB-4 >2.67) quadrupled CVD risk (HR >4.5) and (ii) metabolic burden significantly amplified outcomes (HR=8.04 for ≥4 risk factors). While the association remained significant in most studies, rigorous adjustment for metabolic covariates attenuated effects in some analyses, suggesting that MASLD represents both an independent pathogenic factor and a metabolic dysfunction marker.

NAFLD/MASLD is a significant risk factor for adverse cardiovascular outcomes, with the degree of risk strongly graded by the severity of liver fibrosis and metabolic comorbidity. While evidence is predominantly observational, these findings support integrating non-invasive fibrosis assessment into CVD risk stratification. Future research requires randomized trial testing whether MASLD-targeted therapies can reduce cardiovascular events.

## Introduction and background

Non-alcoholic fatty liver disease (NAFLD), now redefined as metabolic dysfunction-associated steatotic liver disease (MASLD), is characterized by hepatic steatosis (≥5% fat accumulation on imaging or biopsy) without secondary causes such as excessive alcohol consumption (>20-30 g/day for men, >10-20 g/day for women) or other liver diseases [1,2]. The updated MASLD criteria further require ≥1 cardiometabolic risk factor (e.g., obesity, diabetes, or hypertension) alongside steatosis [1]. Affecting 25-30% of adults globally, NAFLD/MASLD shares pathophysiological pathways with metabolic syndrome, including insulin resistance, chronic inflammation, and dyslipidemia, which may accelerate atherosclerosis and cardiovascular events (CVEs) [3,4].

While liver biopsy remains the gold standard for diagnosing MASLD (allowing assessment of steatosis, inflammation, and fibrosis), its invasiveness limits widespread use. Non-invasive alternatives include imaging techniques such as magnetic resonance imaging-proton density fat fraction (MRI-PDFF) for assessing hepatic steatosis and transient elastography or magnetic resonance elastography (MRE) for evaluating liver fibrosis, as well as validated serum-based scores like the Fibrosis-4 Index (FIB-4) and NAFLD Fibrosis Score (NFS). These tools, though less precise, are recommended for risk stratification in clinical practice, particularly where biopsy is unavailable [1]. Recent cohort studies demonstrate that NAFLD is associated with a 1.5- to 3-fold increased risk of myocardial infarction (MI), stroke, and cardiovascular mortality, even after adjusting for traditional risk factors [5,6]. For instance, in a retrospective cohort of 11.6 million adults, NAFLD with ≥4 metabolic risk factors conferred a 31-fold higher microvascular risk (hazard ratio (HR) = 31.20) and 8-fold higher macrovascular risk (HR = 8.04) compared to controls [7]. Similarly, fibrosis severity (FIB-4 >2.67) independently predicted a 4.6-fold increase in composite cardiovascular disease (CVD) events [5]. However, heterogeneity exists; some studies report attenuated associations after adjusting for metabolic confounders [8], while others suggest sex-specific risks [9,10]. 

Despite growing evidence, gaps persist. First, the diagnostic criteria for NAFLD/MASLD vary widely (ultrasound, CT, or surrogate scores like fatty liver index (FLI)), potentially misclassifying severity [1,11,12]. Second, most studies are observational, limiting causal inference. Third, the role of NAFLD-specific interventions (for example, fibrosis regression) in CVD prevention remains unclear [13]. 

This systematic review synthesizes recent evidence to evaluate the strength and consistency of the NAFLD-CVD association, addressing methodological disparities and subgroup-specific risks (e.g., by sex, fibrosis stage, or metabolic profile). By focusing on studies with rigorous comparators and adjusted covariates (for example, FIB-4, MetS components), we aim to clarify whether NAFLD is an independent CVD risk factor or a marker of metabolic dysregulation.

## Review

Methods 

We detail our study design, eligibility criteria, search strategy, data extraction, and risk of bias assessment, adhering to PRISMA 2020 guidelines.

*Study Design* 

This systematic review was conducted and is reported in accordance with the Preferred Reporting Items for Systematic Reviews and Meta-Analyses (PRISMA) 2020 statement [14]. The review aimed to investigate the association between NAFLD and cardiovascular outcomes in adults. This systematic review was registered in the International Prospective Register of Systematic Reviews (PROSPERO) under the registration number CRD420251089714.

Eligibility Criteria 

This systematic review included studies focusing on adults (aged 18 years or older) with a diagnosis of NAFLD. NAFLD can be diagnosed using imaging techniques such as ultrasound, MRI-PDFF, vibration-controlled transient elastography (FibroScan), or shear wave elastography. While liver biopsy remains the gold standard for diagnosing NAFLD/MASLD (histologically confirming steatosis, inflammation, and fibrosis) [1,11], validated non-invasive scoring systems, including FIB-4, the NFS, and a FLI score of 30 or higher are increasingly used in clinical practice, particularly where biopsy is unavailable [5,12]. The primary exposure of interest was the presence of NAFLD.

To be eligible, studies must have included a comparison group of individuals without NAFLD, allowing for an assessment of differential outcomes. The outcomes of interest were clearly defined CVEs, specifically MI, stroke, heart failure (HF), cardiovascular mortality, and atherosclerosis. Eligible study designs comprised observational studies, including cohort and case-control studies, as well as clinical trials if they addressed the research question. Only studies published in the English language with full-text availability were considered.

Studies were excluded if they involved pediatric populations (under 18 years), were animal or in vitro research, or primarily focused on alcoholic fatty liver disease or other liver conditions that could confound the NAFLD-cardiovascular outcome association. Furthermore, case reports, letters to the editor, editorials, commentaries, cross-sectional studies (unless providing clear longitudinal data on outcomes), and conference abstracts without sufficient data for extraction were excluded. Studies lacking a clear control or comparison group of individuals without NAFLD, or those that did not report specific clinical cardiovascular outcome data as defined, were also ineligible for inclusion. Systematic reviews and meta-analyses identified during the search were used for background information and identifying potential primary studies but were not themselves included in the primary data synthesis of this review. 

Information Sources and Search Strategy

A comprehensive literature search was performed to identify relevant studies published up to May 20, 2025. The following electronic databases were searched: PubMed/MEDLINE, ScienceDirect, Europe PMC, and the Cochrane Library (specifically for trials). 

The search strategy combined keywords and Medical Subject Headings (MeSH terms where applicable) related to "NAFLD" (e.g., "Non-alcoholic Fatty Liver Disease," "Fatty Liver," "hepatic steatosis") and "cardiovascular outcomes" (e.g., "Cardiovascular Diseases," "Myocardial Infarction," "Stroke," "cardiovascular mortality"). No date restrictions were applied during the initial search, but publication date filters were applied according to specific database functionalities or during the screening process to align with contemporary evidence. Language was restricted to English. The detailed search strategy for each database is presented in Table 1.

**Table 1:** Search Strategy

#	Search Strategy	Databases/Registers Searched	Filters (I/E Criteria) Used	Number of Papers Identified/After Applying I/E Criteria	Date Search Was Done
1	("Non-alcoholic Fatty Liver Disease"[MeSH] OR "Fatty Liver"[MeSH] OR NAFLD OR "nonalcoholic fatty liver" OR "hepatic steatosis") AND ("Cardiovascular Diseases"[MeSH] OR "Myocardial Infarction"[MeSH] OR "Coronary Artery Disease"[MeSH] OR "Stroke"[MeSH] OR "Heart Failure"[MeSH] OR "Atherosclerosis"[MeSH] OR "cardiovascular mortality" OR "CV outcomes" OR "cardiovascular events")	PubMed and Medline	Full text, Adaptive Clinical Trial, Clinical Study, Clinical Trial, Controlled Clinical Trial, Equivalence Trial, Evaluation Study, Multicenter Study, Observational Study, Pragmatic Clinical Trial, Randomized Controlled Trial, English, Humans.	271 total results	20 May 2025
2	("NAFLD" OR "nonalcoholic fatty liver") AND ("cardiovascular mortality" OR "MI" OR "CAD")	Science Direct	Article Type: Research Article; Publication Titles: Journal of Hepatology, Nutrition, Metabolism and Cardiovascular Diseases, Clinical Gastroenterology and Hepatology, Atherosclerosis, Gastroenterology, Cellular and Molecular Gastroenterology and Hepatology, Journal of Clinical and Experimental Hepatology, Annals of Hepatology, Metabolism, Journal of Lipid Research; Language: English, Subject Areas: Medicine and Dentistry	363 total results	15 May 2025
3	ABSTRACT:("non-alcoholic fatty liver disease" OR NAFLD OR "hepatic steatosis") AND ("cardiovascular disease" OR "cardiovascular events" OR "MI" OR "CAD" OR "CV outcomes")	Europe PMC	Research Articles Free Full Text	294 total results	16 May 2025
4	#1 (("non-alcoholic fatty liver disease" OR NAFLD OR "hepatic steatosis")):ti,ab,kw; #2 MeSH descriptor: [Non-alcoholic Fatty Liver Disease]; #3 #1 OR #2; #4 (("cardiovascular disease" OR "cardiovascular outcomes" OR "myocardial infarction" OR "coronary artery disease" OR stroke OR "heart failure")):ti,ab,kw; #5 MeSH descriptor: [Cardiovascular Diseases]; #6 #4 OR #5; #7 #3 AND #6	Cochrane Library	English Trials	332 Total Results	16 May 2025

Study Selection Process

All records identified from the database searches were imported into the Rayyan app [15], for duplicate removal and subsequent screening. Following the removal of duplicates, two review authors independently screened the titles and abstracts of the remaining records against the predefined eligibility criteria. Any disagreements encountered during this initial screening phase were resolved through discussion between the two reviewers to reach a consensus. If a consensus could not be achieved through discussion, the senior author was consulted for a final resolution.

The full texts of articles deemed potentially relevant after the title and abstract screening were retrieved. These full-text articles were then independently assessed for final eligibility by the same two review authors. Reasons for excluding studies at the full-text stage were carefully documented. Disagreements at this stage were again resolved through discussion and consensus between the two reviewers, with arbitration by the senior author if necessary.

Data Extraction Process

A standardized data extraction form was developed and piloted by the review team prior to formal data extraction. Two review authors independently extracted relevant data from each study that met the inclusion criteria. The extracted information encompassed study characteristics (such as first author, publication year, study design), participant characteristics (such as age, sex, NAFLD diagnostic criteria and severity), exposure details, comparator group information, definitions and measures of cardiovascular outcomes (including effect estimates like hazard ratios or odds ratios with 95% confidence intervals), and information on confounder adjustment. Any discrepancies identified during the data extraction process were resolved through discussion and consensus between the two extracting authors, with the senior author providing guidance for unresolved issues.

Risk of Bias Assessment

Two reviewers independently evaluated the methodological quality and risk of bias for each included study. For observational cohort and cross-sectional studies, the Newcastle-Ottawa Scale (NOS) was employed to perform this assessment [16]. For any included randomized controlled trials, the Cochrane Risk of Bias 2.0 (RoB 2) tool was applied [17].

Data Synthesis and Analysis

A narrative synthesis was primarily employed to summarize and integrate the findings from the included studies, given the anticipated heterogeneity in study designs, NAFLD diagnostic methods, patient populations, and the specific cardiovascular outcomes reported. The synthesis focused on describing the observed associations between NAFLD and various cardiovascular outcomes. Key characteristics of the included studies and their principal findings concerning cardiovascular outcomes were presented in summary tables. Where feasible, quantitative data, such as reported risk ratios, odds ratios, or hazard ratios with their 95% confidence intervals, were extracted and tabulated.

Results

Our findings highlight the dose-dependent association between NAFLD/MASLD and cardiovascular risk, emphasizing the roles of fibrosis severity and metabolic burden.

Search Results

**Figure 1:**
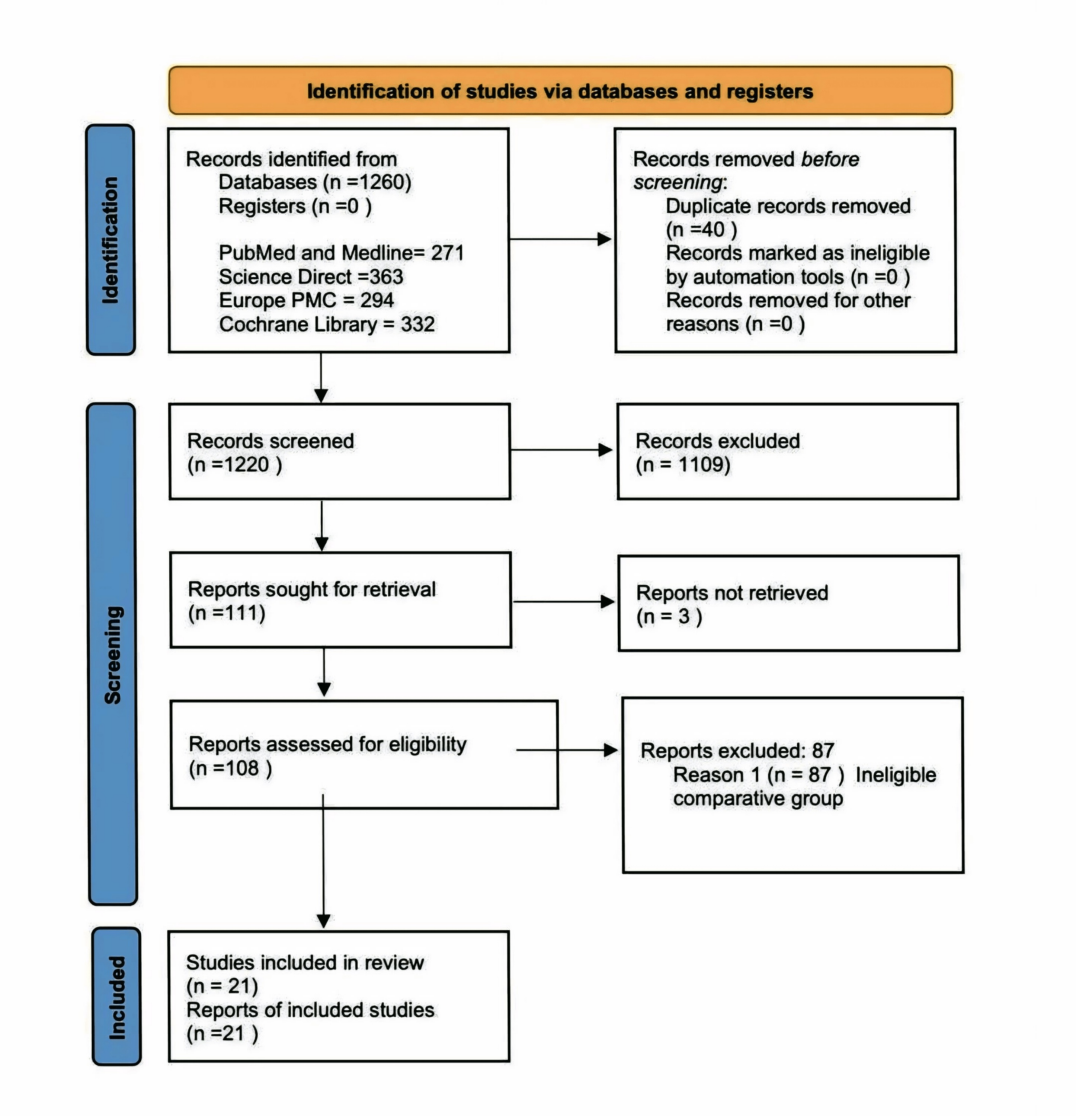
PRISMA Diagram Showing the Article Selection Process PRISMA: Preferred Reporting Items for Systematic Reviews and Meta-Analyses

The study selection process followed the PRISMA 2020 guidelines [14]. Initial database searches identified 1,260 records, with 40 duplicates removed. After screening 1220 titles/abstracts, 1109 records were excluded, leaving 111 for full-text review. Of these, 108 were assessed for eligibility, with 87 excluded due to ineligible comparators (n=87). Ultimately, 21 studies met the inclusion criteria and were incorporated into the systematic review. The PRISMA flow diagram summarizes this process (Figure 1).

Results of Quality Appraisal of Included Studies

The quality of the included studies was evaluated using two distinct instruments: the NOS for the observational studies (comprising 18 cohort and two cross-sectional studies) [16], and the Cochrane RoB 2 tool for the randomized controlled trials (RCTs) [17]. Based on the NOS, studies were categorized as high quality (scores 7-9), moderate quality (scores 4-6), or low quality (scores 0-3). Among NOS-appraised studies, all cohort studies scored ≥7/9 (indicating good quality). Cross-sectional studies scored 7-8/9 (good quality). The RCT was rated to have concerns due to some concerns in missing outcome data (see Table 2 for NOS results and Table 3 for RoB 2 analysis).

**Table 2:** Quality Appraisal of Included Observational Studies Using the Newcastle-Ottawa Scale Selection (maximum of 4 stars), comparability (maximum of 2 stars), outcome (maximum of 3 stars), overall good quality: 7-9 stars NHANES: National Health and Nutrition Examination Survey; UKBB: UK Biobank

Authors (Year)	Study Design	Selection ⭐️ (4)	Comparability ⭐️ (2)	Outcome ⭐️ (3)	Total Score	Overall Quality
Henney et al. (2024) [7]	Prospective cohort	⭐️⭐️⭐️⭐️	⭐️⭐️	⭐️⭐️⭐️	9/9	Good quality
Baratta et al. (2020) [5]	Prospective cohort	⭐️⭐️⭐️⭐️	⭐️⭐️	⭐️⭐️⭐️	9/9	Good quality
Younossi et al. (2022) [2]	Prospective cohort	⭐️⭐️⭐️	⭐️⭐️	⭐️⭐️	7/9	Good quality
Ahmed et al. (2023) [8]	Prospective multi cohort	⭐️⭐️⭐️⭐️	⭐️⭐️	⭐️⭐️⭐️	9/9	Good quality
Nguyen et al. (2021) [18]	Retrospective cohort (NHANES)	⭐️⭐️⭐️⭐️	⭐️⭐️	⭐️⭐️⭐️	9/9	Good quality
Wong et al. (2016) [11]	Prospective cohort	⭐️⭐️⭐️⭐️	⭐️⭐️	⭐️⭐️⭐️	9/9	Good quality
Targher et al. (2007) [3]	Cross-sectional observational	⭐️⭐️⭐️	⭐️⭐️	⭐️⭐️	7/9	Good quality
Song et al. (2025) [19]	Prospective Cohort Study	⭐️⭐️⭐️⭐️	⭐️⭐️	⭐️⭐️⭐️	9/9	Good quality
Meyersohn et al. (2021) [6]	Nested cohort	⭐️⭐️⭐️⭐️	⭐️⭐️	⭐️⭐️⭐️	9/9	Good quality
Lin et al. (2024) [20]	Retrospective cohort (NHANES)	⭐️⭐️⭐️⭐️	⭐️⭐️	⭐️⭐️ ⭐️	9/9	Good quality
Stepanova andYounossi (2012) [21]	Retrospective cohort (NHANES)	⭐️⭐️⭐️⭐️	⭐️⭐️	⭐️⭐️⭐️	9/9	Good quality
Hwang et al. (2018) [9]	Retrospective cohort (Samsung)	⭐️⭐️⭐️ ⭐️	⭐️⭐️	⭐️⭐️⭐️	9/9	Good quality
Kim et al. (2021) [22]	Cohort Study	⭐️⭐️⭐️ ⭐️	⭐️⭐️	⭐️⭐️⭐️	9/9	Good quality
Lee et al. (2021) [23]	Nationwide retrospective cohort	⭐️⭐️⭐️⭐️	⭐️⭐️	⭐️⭐️⭐️	9/9	Good quality
Zhang et al. (2024) [24]	Retrospective cohort (NHANES)	⭐️⭐️⭐️⭐️	⭐️⭐️	⭐️⭐️ ⭐️	9/9	Good quality
Yoo et al. (2023) [25]	Retrospective cohort (Korean)	⭐️⭐️⭐️⭐️	⭐️⭐️	⭐️⭐️ ⭐️	9/9	Good quality
Ren and Zheng (2023) [10]	Retrospective cohort (NHANES)	⭐️⭐️⭐️⭐️	⭐️⭐️	⭐️⭐️⭐️	9/9	Good quality
Lin et al. (2024) [20]	Prospective cohort	⭐️⭐️⭐️⭐️	⭐️⭐️	⭐️⭐️⭐️	9/9	Good quality
Mellinger et al. (2015) [26]	Cross-sectional (Framingham)	⭐️⭐️⭐️⭐️	⭐️⭐️	⭐️⭐️	8/9	Good quality
Jin et al. (2025) [12]	Retrospective cohort (UKBB)	⭐️⭐️⭐️⭐️	⭐️⭐️	⭐️⭐️⭐️	9/9	Good quality

**Table 3:** Quality Appraisal of a Randomised Controlled Trial Study, Using the Cochrane Risk of Bias 2 Tool Randomised Controlled Trial Study from reference [13]

Risk of Bias Domain	Judgment	Support for Judgment
🟢 D1. Bias due to randomization	Low risk	Adequate random sequence generation and allocation concealment were reported.
🟢 D2. Bias due to deviations from interventions	Low risk	Blinding of participants and personnel was ensured; minimal deviations were observed.
🟡 D3. Bias due to missing outcome data	Some concerns	Moderate attrition was noted, though balanced and handled with appropriate statistical methods.
🟢 D4. Bias in measurement of the outcome	Low risk	Pre-specified objective outcome measures were used consistently across groups.
🟢 D5. Bias in selection of the reported result	Low risk	The protocol was available and followed; all outcomes were reported as planned.
🟡 Overall Risk of Bias	Some concerns	Some concerns were due to missing outcome data, despite low risk in other domains.

Characteristics of Included Studies

The included studies encompassed a wide range of populations and settings. Several were conducted within large, general population cohorts, such as the National Health and Nutrition Examination Survey (NHANES) in the US [18,21], the UK Biobank and nationwide health screening databases in Korea [23]. Other studies focused on specific high-risk clinical populations, including patients with established type 2 diabetes mellitus (T2DM) [3], those with known coronary heart disease [19], or individuals undergoing coronary angiography [2,11]. The sample sizes varied dramatically, from several hundred participants in clinical cohorts to over 9.5 million in a nationwide database study. 

The definition of the exposure-fatty liver disease varied across the studies, reflecting the evolution of diagnostic criteria over time. Most studies relied on non-invasive methods. Ultrasound was a common modality for diagnosing NAFLD [5,11], while others used computed tomography (CT) [6,8] or validated non-invasive indices such as the FLI or triglyceride-glucose (TyG) index [19,23]. More recent studies adopted the newer MAFLD or MASLD terminology, which incorporates metabolic risk factors directly into the definition [7,12].

The primary comparison group in most studies consisted of individuals without fatty liver disease, as determined by the same diagnostic method used for the exposure group. Several studies also conducted internal comparisons between different subgroups, such as NAFLD vs. MAFLD [5,18] or by severity of liver fibrosis using scores like FIB-4 [5].

The outcomes reported were consistent with the review's objectives, focusing on CVEs and mortality. These included composite endpoints like major adverse cardiovascular events (MACEs) or major adverse cardiac and cerebrovascular events (MACCEs), as well as specific outcomes such as MI, stroke, HF, and cardiovascular or all-cause mortality. Some studies also reported on the prevalence or severity of coronary artery disease (CAD) as a key outcome.

The characteristics of each study, including population, exposure, and comparison group definitions, and outcomes reported, are detailed in Table 4.

**Table 4:** Characteristics of Included Studies AAC: Abdominal Aortic Calcification; aHR: Adjusted Hazard Ratio; AF: Atrial Fibrillation; AFLD: Alcohol-associated Fatty Liver Disease; ALT: Alanine Aminotransferase; ALD: Alcohol-related Liver Disease; AP: Alkaline Phosphatase; ASCVD: Atherosclerotic Cardiovascular Disease; AST: Aspartate Aminotransferase; BMI: Body Mass Index; CAC: Coronary Artery Calcification; CAD: Coronary Artery Disease; CARDIA: Coronary Artery Risk Development in Young Adults; CHD: Coronary Heart Disease; CI: Confidence Interval; CT: Computed Tomography; CVD: Cardiovascular Disease; CVE: Cardiovascular Event; EF: Ejection Fraction; EAT: Epicardial Adipose Tissue; FHS: Framingham Heart Study; FIB-4: Fibrosis-4 Index; FLD: Fatty Liver Disease; FLI: Fatty Liver Index; GGT: Gamma-glutamyl Transferase; HCC: Hepatocellular Carcinoma; HF: Heart Failure; HR: Hazard Ratio; HSI: Hepatic Steatosis Index; HU: Hounsfield Unit; ICD-10: International Classification of Diseases, 10th Revision; IDF: International Diabetes Federation; LDL-C: Low-Density Lipoprotein Cholesterol; LFTs: Liver Function Tests; LVEDP: Left Ventricular End-Diastolic Pressure; MACE: Major Adverse Cardiovascular Events; MAFLD: Metabolic Dysfunction-Associated Fatty Liver Disease; MD: Metabolic Dysfunction; MetS: Metabolic Syndrome; MI: Myocardial Infarction; NAFLD: Non-alcoholic Fatty Liver Disease; NFS: NAFLD Fibrosis Score; NHANES: National Health and Nutrition Examination Survey; NLR: Neutrophil-to-Lymphocyte Ratio; NS: Not Significant; OR: Odds Ratio; PAD: Peripheral Artery Disease; PCI: Percutaneous Coronary Intervention; PLR: Platelet-to-Lymphocyte Ratio; Q1, Q4: Quartile 1, Quartile 4; RF: Risk Factor; SII: Systemic Immune-Inflammation Index; SIRI: Systemic Inflammatory Response Index; SLD: Steatotic Liver Disease; T2DM: Type 2 Diabetes Mellitus; TIA: Transient Ischemic Attack; TVR: Target Vessel Revascularization; TyG: Triglyceride-Glucose Index; US FLI: Ultrasound Fatty Liver Index

Study ID	Population	Exposure Definition	Comparison Group	Outcomes Reported
Henney et al. (2024) [7]	Retrospective cohort from the TriNetX network (N = 11,694,246 reference; 462,789 MASLD + 4 RFs)	MASLD: Hepatic steatosis (ICD-10 codes or modified HSI) + ≥1 MetS component (obesity, insulin resistance, hypertension, dyslipidemia). Subgroups: MASLD + 1 to 4 RFs.	Adults without hepatic steatosis or any MetS components (reference arm).	Microvascular disease: Peripheral neuropathy, retinopathy, nephropathy (HR = 31.20 for MASLD + 4 RFs). Macrovascular disease: CVD events, cerebrovascular accidents, peripheral vascular disease (HR = 8.04 for MASLD + 4 RFs).
Baratta et al. (2020) [5]	898 consecutive outpatients (mean age 56.4 ± 12.7 years; 37.5% women) with cardiometabolic risk factors (hypertension, obesity, diabetes, dyslipidemia, AF, or MetS).	NAFLD: Ultrasound-confirmed hepatic steatosis (Hamaguchi criteria) + exclusion of other liver diseases/alcohol abuse. Fibrosis: FIB-4 >2.67 or NFS >0.676.	Patients without NAFLD (n = 255) vs. with NAFLD (n = 643). Subgroups by fibrosis severity (FIB-4/NFS thresholds).	Primary: Composite CVEs (fatal/nonfatal ischemic stroke/MI, revascularization, new-onset AF, cardiovascular death). Incidence: 1.9%/year overall; 2.1%/year in NAFLD vs. 1.0%/year in non-NAFLD (P = .066; significant after excluding AF: P = .034). HRs: NAFLD (adjusted HR = 2.73); FIB-4 >2.67 (HR = 4.57); NFS >0.676 (HR = 2.29).
Younossi et al. (2022) [2]	619 patients (mean age 63 ± 10 years; 80% male; 65% NAFLD) undergoing elective cardiac angiography	NAFLD: Hepatic steatosis by ultrasound + exclusion of other liver diseases (alcoholic, viral hepatitis, etc.).	Patients without NAFLD undergoing elective cardiac angiography	Severe CAD (>70% stenosis in ≥1 proximal artery). Risk of severe CAD (angiographic CAD or ASCVD score ≥20). Cardiac dysfunction (elevated LVEDP ≥12 mmHg or EF ≤45%).
Ahmed et al. (2023) [8]	10,040 participants from 3 US cohorts (FHS, CARDIA, MESA; mean age 51.3 ± 3.3 years; 50.6% female; 58.5% non-Hispanic White)	NAFLD: CT-defined hepatic steatosis (liver attenuation <51 HU or liver phantom ratio <0.33); moderate-to-severe: <40 HU.	Participants without hepatic steatosis on CT	Incident CVD (MI, stroke, HF, etc.); CVD mortality; all-cause mortality; incident cancer (excl. nonmelanoma skin cancer)
Nguyen et al. (2021) [18]	2,997 participants from NHANES III (1988–1994; 50.6% female; 73.6% non-Hispanic White; mean age not specified)	NAFLD: Ultrasound-detected steatosis + absence of alcohol/viral hepatitis MAFLD: Steatosis + metabolic dysfunction (T2DM, obesity, or ≥2 metabolic abnormalities) Groups: Non-MAFLD NAFLD, NAFLD-MAFLD, non-NAFLD MAFLD (incl. alcohol/viral hepatitis)	No direct comparator; focus on group comparisons	All-cause mortality highest in non-NAFLD MAFLD (26.2%) vs. 10.6% in non-MAFLD NAFLD; CVD mortality higher in non-NAFLD MAFLD; other-cause mortality (e.g., liver-related) also higher in non-NAFLD MAFLD; cancer mortality not significantly different
Wong et al. (2016) [11]	612 consecutive adults (≥18 years) undergoing coronary angiography in Hong Kong; excluded: alcohol abuse, viral hepatitis, unstable/emergency cases	NAFLD: Diagnosed via abdominal ultrasound (criteria: diffuse liver echogenicity, vascular blurring, deep attenuation); 94% sensitivity, 97% specificity for ≥30% steatosis	Non-NAFLD (no steatosis on ultrasound)	Primary: All-cause mortality (lower in NAFLD: 13.2% vs. 23.0%; aHR 0.36, 95% CI 0.18–0.70). Secondary: Composite CVD events (36.5% vs. 37.1%; aHR 0.90), two liver-related events (HCC) in the NAFLD group
Targher et al. (2007) [3]	2,839 type 2 diabetic outpatients (aged 40–86 years) in Italy; excluded: alcohol >20 g/day, viral hepatitis, other liver diseases	NAFLD: Diagnosed by ultrasound (sonographic features) after excluding other liver conditions; ~90% sensitivity and ~95% specificity for moderate/severe steatosis	Non-NAFLD (no steatosis on ultrasound, normal liver tests)	CVD prevalence higher in NAFLD: coronary (26.6% vs. 18.3%), cerebrovascular (20.0% vs. 13.3%), peripheral (15.4% vs. 10.0%) Adjusted OR for CVD: 1.84 (95% CI 1.4–2.4) after adjusting for metabolic syndrome and confounders
Song et al. (2025) [19]	2,273 CHD patients post-PCI with LDL-C <1.8 mmol/L in China (2013–2013); excluded: missing labs or LDL-C ≥1.8 mmol/L; 800 MAFLD vs. 800 non-MAFLD via propensity score matching	MAFLD: Hepatic steatosis defined by TyG index ≥8.5 + metabolic risk (BMI ≥23 kg/m², T2DM, or ≥2 metabolic abnormalities) per international consensus	Non-MAFLD (TyG <8.5 and no metabolic risks)	Primary: MACCEs (death, MI, stroke, stent thrombosis, TVR). Secondary: All-cause death, MI, stent thrombosis (especially in FIB-4-stratified MAFLD groups)
Meyersohn et al. (2021) [6]	3,756 symptomatic outpatients without known CAD (mean age 60.6 years; 48.4% male) from the PROMISE trial	Hepatic steatosis diagnosed via non-contrast CT: liver attenuation <40 HU, liver-spleen difference <1 HU, or liver-to-spleen ratio <1	2,797 without steatosis vs. 959 with steatosis	Primary outcome: MACE (death, nonfatal MI, unstable angina hospitalization); MACE incidence: 4.4% in steatosis vs. 2.6% without Adjusted HR: 1.69 (95% CI: 1.16–2.48)
Lin et al. (2024) [20]	3,040 MAFLD adults from NHANES III (mean age 56.92 years; 50.6% male)	Serum carotenoids (α-carotene, β-carotene, β-cryptoxanthin, lycopene, lutein/zeaxanthin) measured via HPLC	Quartile comparison (Q1 vs. Q4)	All-cause mortality: 1,325 deaths; cardiovascular mortality: 429 deaths (ICD-10 I00–I09, I11, I13, I20–I69); HRs for Q4 vs. Q1: α-carotene 0.63, β-carotene 0.65, β-cryptoxanthin 0.64, lycopene 0.73, lutein/zeaxanthin 0.69 (all-cause); α-carotene 0.51, β-carotene 0.54, β-cryptoxanthin 0.52, lycopene 0.63, lutein/zeaxanthin 0.62 (CVD)
Stepanova and Younossi (2012) [21]	NHANES III (N=11,613; age 20–74 years)	NAFLD: Ultrasound-confirmed moderate/severe hepatic steatosis + exclusion of excessive alcohol, viral hepatitis, iron overload. Subgroups by elevated vs. normal liver enzymes (ALT/AST).	Non-NAFLD controls (no steatosis or liver disease)	CVD prevalence: 38.2% in NAFLD vs. 29.3% controls (OR = 1.23, 95% CI 1.04–1.44); cardiovascular mortality: 5.6% vs. 3.3% (not significant after adjustment); no independent association of NAFLD with CV mortality
Hwang et al. (2018) [9]	Kangbuk Samsung Health Study cohort (N=318,224; age 20–94 years, mean 39.3)	NAFLD: Ultrasound-confirmed hepatic steatosis; exclusion of excess alcohol/viral hepatitis. Subgroups: normal vs. elevated liver enzymes	Non-NAFLD controls (no steatosis, or liver disease)	All-cause mortality: 0.60% in NAFLD vs. 0.47% controls; cardiovascular mortality: 0.11% NAFLD vs. 0.07% controls (HR=1.63 women; NS men); cancer mortality: lower in men with NAFLD (HR=0.79), higher in women (HR=1.83); liver mortality: 5.58x higher in women
Kim et al. (2021) [22]	NHANES III cohort (N = 7,761; age 20–74 years)	NAFLD: Ultrasound-confirmed steatosis without excessive alcohol/viral hepatitis. MAFLD: Steatosis + ≥1 metabolic risk factor (obesity, diabetes, or ≥2 metabolic abnormalities).	Non-NAFLD/ non-MAFLD controls (no steatosis on ultrasound)	All-cause mortality: HR=1.33 for MAFLD; HR=1.17 for NAFLD (NS after metabolic adjustment); Cardiovascular mortality: HR=1.24 for MAFLD (NS after adjustment); Cancer mortality: HR=1.95 for MAFLD(+)/NAFLD(–) subgroup.
Lee et al. (2021) [23]	9,584,399 middle-aged Korean adults (40–64 years; 48.5% male) from nationwide health screening database (2009–2010). Longitudinal: 8,962,813 participants	NAFLD: Hepatic steatosis (FLI ≥30) + no excessive alcohol (>30 g/day men; >20 g/day women) or other liver disease. MAFLD: Steatosis + ≥1 metabolic risk factor (BMI >23, diabetes, or ≥2 metabolic abnormalities).	Neither FLD (no NAFLD or MAFLD). NAFLD-only (NAFLD but not MAFLD). MAFLD-only (MAFLD but not NAFLD). Both FLD (both definitions).	Primary: Composite CVD events (MI, ischemic stroke, heart failure hospitalization, CVD death). Secondary: Individual CVD events. HRs vs. Neither FLD: Both-FLD 1.56, MAFLD-only 1.43, NAFLD-only 1.09.
Zhang et al. (2025) [24]	4,787 MASLD patients from NHANES (2005–2018; mean age 51.9; 56.7% male)	MASLD: Hepatic steatosis (US FLI ≥30) + ≥1 cardiometabolic risk factor; excludes other liver diseases or excess alcohol (>3 drinks/day men; >2 women).	Quartiles of systemic inflammation markers (SII, SIRI, NLR, PLR) and cutoff-based high/low groups.	Primary: Cardiovascular mortality (174 events). Secondary: All-cause mortality (567 events). HRs for CVD mortality (Q4 vs. Q1): SII 3.22, SIRI 2.74, NLR 3.69, PLR 1.83.
Yoo et al. (2023) [25]	Korean cohort from Kangbuk Samsung Health Study (N=701,664; mean age 39.8±10.9 years; 52.56% male)	NAFLD: Ultrasound-confirmed hepatic steatosis + no excess alcohol or viral hepatitis. MAFLD: Hepatic steatosis + (BMI≥23, diabetes, or ≥2 metabolic abnormalities). AFLD: Hepatic steatosis + excessive alcohol intake.	No FLD or non-MAFLD/non-NAFLD groups	CVD mortality: MAFLD vs no-MAFLD: HR 1.14 (95% CI 1.02–1.28)- NAFLD: NS trend HR 1.07 (0.95–1.21) MAFLD-only subgroup: HR 1.35 (1.07–1.70)
Ren and Zheng (2023) [10]	NHANES 2000–2014 cohort (N=2,627; age ≥18 years; 65.4% male; mean age 48.6 years)	NAFLD: US Fatty Liver Index (USFLI) ≥30; excludes excessive alcohol (>2 drinks/day men, >1 drink/day women) and viral hepatitis	Sex-stratified (male vs female NAFLD patients)	All-cause mortality: higher in males (12.4% vs. 7.7%, p=0.005). CV mortality: higher in females ≤60 years (adjusted HR 0.214; 95% CI 0.053–0.869; p=0.031)
Lin et al. (2021)[4]	EISNER study cohort (N=2,068; mean age 55.6 ± 9.1 years; 59% male; asymptomatic; intermediate CAD risk)	NAFLD: CT-defined (liver-to-spleen ratio <1.0 or liver attenuation <40 HU). MetS: IDF criteria (BMI ≥30 + ≥2 metabolic abnormalities). EAT: AI-quantified volume/attenuation from CAC-CT.	Participants without MetS/NAFLD or with low EAT volume (<113 cm³)	MACE (MI, late revascularization, cardiac death) incidence 10.8%. Adjusted HRs: MetS 1.58 (95% CI 1.10–2.27), NAFLD 1.78 (95% CI 1.21–2.61).
Athyros et al. (2011) [13]	1,123 patients with MetS and abnormal LFTs, aged 45–65; NAFLD subgroup n=326 (ultrasound-confirmed)	NAFLD: Elevated LFTs + ultrasound hepatic steatosis. MetS: AHA criteria (≥3 of high TG, low HDL-C, hypertension, abdominal obesity, high fasting glucose).	Two treatment groups by LDL-C targets: Group A2: <100 mg/dl (n=165) Group B2: <130 mg/dl (n=161)	NAFLD resolution: 86% in Group A2 vs 74% in Group B2 (p<0.001). CVD events: 0 in Group A2 vs 5 non-fatal in Group B2 (p=0.024). Greater LFT normalization in Group A2 (ALT, AST, γ-GT, AP; p<0.05).
Mellinger et al. (2015) [26]	Framingham Heart Study participants (N=3,014; mean age 51.1 ±10.1 years; 50.5% women); hepatic steatosis prevalence 17%	Hepatic steatosis measured by multidetector CT (liver-phantom ratio ≤0.33 = ≥30% steatosis; 98% sensitivity, 70% specificity), adjusted for alcohol use	Participants without hepatic steatosis (liver-phantom ratio >0.33)	Clinical CVD (non-fatal MI, stroke, TIA, heart failure, PAD): OR 1.14, p=0.07 (not significant). Subclinical CVD: CAC (OR 1.20, p<0.001), AAC (OR 1.16, p<0.001; stronger in men, p-interaction=0.022)
Jin et al. (2025) [12]	UK Biobank cohort (N = 340,998; age 40–69 years). Subgroups: MAFLD (n=126,077; 36.97%), MASLD (n=97,418; 28.57%)	MAFLD: Fatty Liver Index (FLI ≥60) plus overweight/obesity, diabetes, or ≥2 metabolic dysfunctions (MD). MASLD: FLI ≥60 plus ≥1 cardiometabolic risk factor (excluding excess alcohol). Subtypes: MAFLD (diabetes, overweight ± MD, lean metabolic disorder), SLD (MASLD, MetALD, ALD, cryptogenic).	Non-MAFLD/non-MASLD (FLI <60 + no metabolic risk factors)	CVD events (CAD, stroke, HF, CVD death): HR 1.52 for MAFLD, 1.42 for MASLD. Highest risk in MAFLD diabetes subtype (HR 2.26) and ALD subtype (HR 1.65). Overweight MAFLD without MD showed no increased CVD risk.

Synthesis of Key Findings of Included Studies

The follow-up duration across the longitudinal studies varied considerably, ranging from a median of 24 months [19] to 25.8 years [20], while several studies employed a cross-sectional design with no follow-up. All observational studies with longitudinal follow-up used multivariable statistical models to adjust for potential confounders, most commonly including age, sex, BMI, smoking status, and key metabolic risk factors such as diabetes and hypertension.

A primary finding across multiple large cohort studies was a statistically significant association between NAFLD/MASLD and an increased risk of incident MACEs. Meyersohn et al. (2021) reported that hepatic steatosis was independently associated with a 69% higher risk of MACE (adjusted HR = 1.69) [6], while Baratta et al. (2020) found that NAFLD conferred a more than two-fold increased risk of composite CVEs (adjusted HR = 2.73) [5]. This finding was not universal; however, as noted by Ahmed et al. (2023), the association with incident CVD became non-significant after adjustment for time-varying metabolic covariates [8], while Wong et al. (2016) similarly found no significant difference in composite CVD outcomes in their angiography cohort [11]. The relationship with mortality was similarly complex. While some studies linked certain MAFLD subtypes to significantly higher all-cause mortality (HR = 2.4) [18], others found that any association with cardiovascular mortality specifically was attenuated and lost statistical significance after full adjustment for metabolic factors [21,22].

A consistent theme emerging from the evidence was that cardiovascular risk is substantially amplified by the presence of advanced liver fibrosis and the burden of metabolic dysfunction. For instance, Baratta et al. (2020) demonstrated that a FIB-4 score >2.67 increased CVE risk by over four-fold (HR = 4.57), a much stronger association than for NAFLD alone [5]. Similarly, Song et al. (2025) reported that MAFLD patients with a high FIB-4 score had a nearly five-fold higher risk of subsequent MI [19]. The importance of metabolic health was further highlighted in studies directly comparing diagnostic criteria [19]. Both Lee et al. (2021) and Jin et al. (2025) reported that the newer MAFLD definition [12,23], which explicitly incorporates metabolic risk, was a stronger predictor of CVD events than the traditional NAFLD definition. The key findings and methodological characteristics of the 21 included studies are detailed in Table 5.

**Table 5:** Summary of Key Findings and Methodological Characteristics of Included Studies AAC: Abdominal Aortic Calcification; AF: Atrial Fibrillation; AFLD: Alcohol-Associated Fatty Liver Disease; ALT: Alanine Aminotransferase; AST: Aspartate Aminotransferase; AUC: Area Under the Curve; BMI: Body Mass Index; BP: Blood Pressure; CAC: Coronary Artery Calcification; CAD: Coronary Artery Disease; CI: Confidence Interval; CKD: Chronic Kidney Disease; CVD: Cardiovascular Disease; CVEs: Cardiovascular Events; DM: Diabetes Mellitus; EF: Ejection Fraction; eGFR: Estimated Glomerular Filtration Rate; FIB-4: Fibrosis-4 Index; FLD: Fatty Liver Disease; FLI: Fatty Liver Index; HbA1c: Hemoglobin A1c; HDL: High-Density Lipoprotein; HR: Hazard Ratio; HSI: Hepatic Steatosis Index; IQR: Interquartile Range; LDL-C: Low-Density Lipoprotein Cholesterol; LFT: Liver Function Tests; MACCE: Major Adverse Cardiac and Cerebrovascular Events; MAFLD: Metabolic Dysfunction-Associated Fatty Liver Disease; MASLD: Metabolic Dysfunction-Associated Steatotic Liver Disease; MACE: Major Adverse Cardiovascular Events; MetS: Metabolic Syndrome; MI: Myocardial Infarction; MRI: Magnetic Resonance Imaging; NAFLD: Non-Alcoholic Fatty Liver Disease; NASH: Non-Alcoholic Steatohepatitis; NFS: NAFLD Fibrosis Score; NHANES: National Health and Nutrition Examination Survey; NLR: Neutrophil-to-Lymphocyte Ratio; NT-proBNP: N-terminal pro–B-type Natriuretic Peptide; OR: Odds Ratio; PSM: Propensity Score Matching; RCT: Randomized Controlled Trial; SIRI: Systemic Inflammatory Response Index; SLD: Steatotic Liver Disease; T2DM: Type 2 Diabetes Mellitus; TyG: Triglyceride–Glucose Index; USFLI: Ultrasound Fatty Liver Index

Study ID	Subgroups Analyzed	Follow-up Duration	Adjusted Covariates	Key Findings	Limitations
Henney et al. (2024) [7]	MASLD + individual MetS components (e.g., insulin resistance, hypertension). MASLD + cumulative RFs (1 to 4). Sex-stratified analysis (higher risk in women).	5 years	Propensity score matching (1:1) for age, sex, ethnicity, smoking, GFR, BMI, HbA1c, blood pressure, triglycerides.	Insulin resistance conferred the highest microvascular risk (HR = 13.93). Hypertension conferred the highest macrovascular risk (HR = 7.23). Cumulative MetS components increased risk (HR = 31.20 for microvascular, 8.04 for macrovascular with 4 RFs). Women had a higher risk than men.	Retrospective design (potential residual confounding). Underdiagnosis of MASLD (used ICD-10/modified HSI). No liver biopsy/fibrosis staging. Alcohol consumption not fully excluded (only ICD-10 codes).
Baratta et al. (2020) [5]	≤2.67 vs. >2.67; NFS ≤0.676 vs. >0.676). Excluded new-onset AF in sensitivity analysis. Patients without prior CVEs at baseline.	Median 41.4 months (IQR: 23.2–62.8); 3044.4 person-years	Model A (NFS): Age, sex, prior CVEs, statin use, hypertension, MetS. Model B (FIB-4): Age, sex, MetS, Hamaguchi score.	NAFLD independently increased CVE risk (HR = 2.73). Fibrosis (FIB-4 >2.67) conferred 4.6-fold higher CVE risk; NFS >0.676 doubled risk. MetS, male sex, and prior CVEs were additional predictors. Results robust after excluding AF or patients with prior CVEs.	Diagnostic: Ultrasound (not biopsy/MRI) for steatosis; FIB-4/NFS as surrogate fibrosis markers. Design: Observational (no causality); single-center; small CVE numbers for subtype analysis. Confounding: Lack of data on aspirin use, familial CVD history, or carotid plaques.
Younossi et al. (2022) [2]	Patients with NAFLD vs. without NAFLD. Patients with severe CAD vs. no severe CAD. Patients with risk of severe CAD vs. no risk.	Cross-sectional study (no follow-up)	Age, gender, MetS components (diabetes, hyperlipidemia, hypertension), statin use, smoking, hs-cTnI, NT-proBNP.	44% of NAFLD patients had severe CAD; 58% had a risk of severe CAD. hs-cTnI independently associated with severe CAD (OR 2.01, CI 1.37–2.93) and risk of CAD (OR 1.84, CI 1.14–2.96). NT-proBNP associated with cardiac dysfunction (elevated LVEDP/low EF). No link between liver fibrosis scores and CAD outcomes.	Referral bias (high CAD prevalence in angiography cohort). No longitudinal data. Missing confounders (physical activity, nutrition). Ultrasound NAFLD diagnosis is less sensitive than biopsy/MRI.
Ahmed et al. (2023) [8]	Mild vs. moderate-to-severe steatosis Stratified by age, sex, metabolic risk factors	Mean 12.7 years (127,481 person-years)	Baseline models: Age, sex, diabetes, systolic BP, alcohol, smoking, HDL, TGs, BMI. Time-varying models: Updated covariates	Hepatic steatosis linked to all-cause mortality (HR: 1.21; 95% CI: 1.04–1.40) in baseline but not in time-varying models. No significant link with incident CVD or CVD mortality after adjusting for BMI/time-varying covariates. No link with incident cancer. Associations attenuated after accounting for metabolic risk, suggesting cardiometabolic confounding.	Referral bias: Not generalizable. Few events: Low CVD/cancer case numbers due to a younger cohort. CT limits: Can’t assess NASH/fibrosis. No time-updated liver fat: Only baseline liver fat assessed.
Nguyen et al. (2021) [18]	Non-MAFLD NAFLD (8.5%); NAFLD-MAFLD (74.7%) Non-NAFLD MAFLD (16.8%); Stratified by alcohol use and sex	Up to 27 years (till 2015)	Age, sex, race, smoking, viral hepatitis, FIB-4, weight class, diabetes, hypertension	Non-NAFLD MAFLD had the highest mortality risk (HR 2.4, 95% CI 1.2–4.6 vs. non-MAFLD NAFLD). Advanced fibrosis more common in non-NAFLD MAFLD (7.5%) vs. NAFLD-MAFLD (1.3%). Black race linked to higher all-cause mortality (HR 1.3). No significant cancer mortality differences	Ultrasound limits: Less accurate than MRI/biopsy. Self-reported alcohol/comorbidity data. No liver-specific mortality data (NHANES restriction). Underrepresentation: Lacked Asian/other minorities
Wong et al. (2016) [11]	Patients with or without coronary artery stenosis (>50%)	Median 72 months (6 years)	Age, sex, BMI, smoking, diabetes, hypertension, statin use, coronary interventions (medical, PCI, CABG)	Higher baseline CAD in NAFLD (84.6% vs. 64.1%) and more interventions (68.3% vs. 43.4%). Lower all-cause mortality in NAFLD group (fewer CVD deaths). No significant difference in composite CVD outcomes. Liver-related events rare (0.6% in NAFLD group)	Ultrasound may miss mild steatosis. Treatment effects (medications, revascularization) may confound results. Short follow-up for detecting liver outcomes. No fibrosis staging, so NAFLD severity unclear
Targher et al. (2007) [3]	By age (40–59 vs. ≥60), and by sex (NAFLD in 71.1% men vs. 68.0% women)	Cross-sectional (no follow-up)	Age, sex, BMI, smoking, diabetes duration, A1C, LDL, use of hypoglycemic, antihypertensive, lipid-lowering, antiplatelet meds, ATP III metabolic syndrome	NAFLD prevalence: 69.5% in diabetics NAFLD significantly linked to higher coronary, cerebrovascular, and peripheral CVD (P < 0.001). Association independent of metabolic syndrome (OR 1.84). 86% of NAFLD patients had normal ALT, highlighting limits of liver enzyme screening	Cross-sectional: cannot determine causality Ultrasound limitations: can’t assess fibrosis or distinguish. NASH: No liver biopsy or insulin resistance data. Mild steatosis may be missed on ultrasound
Song et al. (2025) [19]	Age (<60 vs. ≥60), sex, diabetes, hyperlipidemia, hypertension; MAFLD by fibrosis (FIB-4 <3.25 vs. ≥3.25)	24 months (median not specified)	PSM balanced for age, sex, smoking, MI history, cardiac/renal dysfunction, CAD severity, hypertension, hyperlipidemia, T2DM; Cox models adjusted for residual confounders	MAFLD linked to 48% higher MACCE risk vs. non-MAFLD (HR 1.48). FIB-4 ≥3.25: 2.91× higher MACCEs, 4.79× higher death risk, 4.98× higher MI risk. Risk consistent across subgroups (age, sex, comorbidities)	Retrospective, single-center study. FIB-4 used for fibrosis (not biopsy). Short two-year follow-up. TyG-based steatosis assessment less validated than imaging.
Meyersohn et al. (2021) [6]	Coronary artery disease severity (none, non-obstructive, stenosis); metabolic syndrome and obesity subgroups	Median 25 months	ASCVD risk score, >70% coronary stenosis, metabolic syndrome, obesity, CT-based plaque metrics (segment involvement, Leaman score, Agatston score)	Steatosis independently linked to 69% higher MACE risk. Risk persisted after adjusting for plaque burden and metabolic factors. Risk prediction improved with steatosis: net reclassification index = 0.24	No histological confirmation of liver fat. Alcohol use and HCV status not assessed. Short follow-up (25 months). CT may miss mild steatosis.
Lin et al. (2024) [20]	By quartiles of each carotenoid and total serum carotenoids	Median 25.8 years (826,547 person-years)	Model 1: Age, sex, race, education, income. Model 2: + smoking, alcohol, BMI, supplements. Model 3: + triglycerides, CRP, diabetes	Higher serum carotenoids linked with lower all-cause and CVD mortality. HRs ranged from 0.51 to 0.73 in Q4 vs. Q1; Linear dose-response for α-carotene, β-cryptoxanthin, and lycopene nonlinear for β-carotene and lutein/zeaxanthin. Findings consistent across adjusted models	Observational design limits causal inference. Single baseline carotenoid measurement. MAFLD defined by ultrasound (no biopsy). No data on MAFLD severity. Limited to U.S. population (generalizability concern)
Stepanova and Younossi (2012) [21]	NAFLD with elevated enzymes - NAFLD with normal enzymes - Age groups: 35–44, 45–54, 55–64, 65–74 years	Median 171 months (~14 years)	Age, sex, race, obesity, diabetes, smoking, family history of CVD, and insulin resistance	NAFLD independently associated with increased CVD risk (OR 1.23). No independent link between NAFLD and CV mortality. NAFLD patients had more metabolic syndrome features. CVD was the leading cause of death (5.6%)	NAFLD diagnosis by ultrasound (no biopsy/MRI). Limited mortality follow-up. No liver-specific mortality data. Cross-sectional CVD assessment (self-report)
Hwang et al. (2018) [9]	By sex (men vs. women). By obesity status (BMI ≥25). By liver fibrosis (FIB-4 ≥1.3)	Median 5.7 years	Age, BMI, smoking, alcohol, physical activity, diabetes, hypertension, and hypercholesterolemia	NAFLD linked to increased all-cause, cancer, CVD, liver mortality in women only (all HRs significant). Men with NAFLD had lower cancer mortality (HR=0.79). Advanced fibrosis (FIB-4≥1.3) raised mortality in both sexes (e.g., HR=4.81 all-cause in men). Nonobese women with NAFLD had higher mortality risk; obese men with NAFLD had lower cancer mortality	Ultrasound diagnosis only (no biopsy/MRI). Short follow-up (5.7 years). Hospital-based cohort (selection bias possible). No data on medications or lifestyle changes
Kim et al. (2021) [22]	Concordant: MAFLD(+)/NAFLD(+) (23.5%). Discordant: MAFLD(+)/NAFLD(–) (metabolic risk + other liver disease; 2.4%), MAFLD(–)/NAFLD(+) (lean NAFLD; 6.1%). Advanced fibrosis: Defined by NFS, FIB-4, APRI	Median 23.2 years (IQR 21.7–25.0)	Model 1: Age, sex, race, education, smoking, ALT, sedentary lifestyle Model 2: Model 1 + BMI, diabetes, hypertension, lipids, CRP	MAFLD linked to 33% higher all-cause mortality (HR=1.33). Discordant MAFLD(+)/NAFLD(–) subgroup had 86% higher mortality risk (HR=1.66). Advanced fibrosis in MAFLD doubled mortality risk (HR=2.00), NAFLD risk attenuated (HR=1.45, NS). NAFLD mortality risk weakened after metabolic adjustment.	Ultrasound diagnosis only (no biopsy/MRI). No liver-specific mortality data (NHANES limitation). Data from 1988–1994 (may not reflect current obesity trends). No reassessment of NAFLD/MAFLD status over time.
Lee et al. (2021) [23]	Sex (male vs. female). Age (<50 vs. ≥50 years). Metabolic risk factors (obesity, diabetes, hypertension, dyslipidemia). Liver disease/alcohol consumption.	Median 10.1 years (until Dec 2019)	Age, sex, household income, residential area, Charlson Comorbidity Index, tobacco use, exercise frequency, and eGFR. Metabolic factors not adjusted in main analysis (intrinsic to MAFLD).	MAFLD prevalence higher than NAFLD (37.3% vs. 28%), especially in men. MAFLD-only and Both-FLD had higher CVD risk (HR 1.43–1.56) than NAFLD-only (HR 1.09). NAFLD-only had lower CVD risk and healthier metabolic profiles. MAFLD-CVD link persisted after stratification.	Steatosis assessed by FLI, not imaging/biopsy. Single baseline assessment. No fibrosis data. Korean cohort; uncertain generalizability.
Zhang et al. (2025) [24]	Age, sex, race, BMI, smoking, diabetes, hypertension, CKD, advanced fibrosis (FIB-4 >2.67).	Median 7.0 years (IQR 3.8–10.3)	Model 1: Age, sex, race, education, BMI, smoking. Model 2: Model 1 + hypertension, diabetes, CVD, cancer, CKD, advanced fibrosis.	SIRI and NLR showed superior prediction of CVD mortality (AUC 0.70 and 0.69) vs. SII and PLR. High inflammation cutoffs: SIRI >1.23 (HR=2.67), NLR >2.18 (HR=2.39) for CVD mortality. Combining SIRI/NLR with FIB-4 improved prediction (AUC=0.80).	Steatosis diagnosis by US FLI, not biopsy/imaging. Single-time inflammation measurement; no longitudinal data. Potential unmeasured confounders (diet, genetics). NHANES U.S. cohort; external validation needed.
Yoo et al. (2023) [25]	5 categories: 1. No FLD (reference) 2. NAFLD-only 3. MAFLD-only 4. Both NAFLD and MAFLD 5. AFLD and non-MAFLD	Median 8.77 years (mean 9.24 ± 5.26 years)	Model 1: Age, sex, education, smoking, exercise. Model 2: Model 1 + LDL cholesterol.	MAFLD associated with 14% higher CVD mortality vs no-MAFLD. NAFLD not significantly linked to CVD mortality. MAFLD-only subgroup had 35% higher CVD mortality. AFLD/non-MAFLD showed elevated but NS risk.	Single ethnicity (Korean), relatively young cohort with low baseline mortality. Ultrasound-based steatosis diagnosis. No accounting for time-varying factors. No dietary or repeated FLD assessments.
Ren and Zheng (2023)[10]	Age (≤60 vs >60), BMI (≤30 vs >30), hypertension/diabetes status	Median 10.83 years	Model 1: BMI. Model 2: BMI + smoking. Model 3: Model 2 + alcohol, hypertension, diabetes. Model 4: Model 3 + ALT, AST, cholesterol, albumin	All-cause mortality higher in males across all ages (HR 2.14 for ≤60, HR 1.60 for >60). CV mortality higher in females ≤60 (HR 0.31, p=0.03). Males with BMI >30 or diabetes had higher mortality (HR 1.85 and 1.68).	USFLI for NAFLD diagnosis (no biopsy/MRI). Self-reported comorbidities. No hormonal or fibrosis biomarkers. Cross-sectional baseline data only.
Lin et al. (2021) [4]	MetS vs no MetS; NAFLD vs no NAFLD; High (≥113 cm³) vs low EAT; CAC score strata (0, 1–100, 101–400, >400)	Mean 14 ± 3 years	Model 1: Age, sex, smoking, LDL, statin use, antihypertensives. Model 2: Model 1 + CAC score. Model 3: Model 2 + EAT volume/attenuation.	MetS, NAFLD, and EAT all independently predicted MACE. EAT volume improved 22% net reclassification index over ASCVD score (p=0.002). NAFLD stronger predictor than MetS after adjusting for EAT/CAC. Worst survival with high EAT + NAFLD (log-rank p<0.001).	CT-defined NAFLD (not biopsy). No waist circumference data for MetS. Mostly Caucasian cohort. No hormonal or fibrosis biomarkers.
Athyros et al. (2011) [13]	NAFLD patients (n=326) stratified by LDL-C targets.	42 months (3.5 years)	Intention-to-treat; ANOVA, t-tests, χ², log-rank test for survival.	Lower LDL-C target group (A2) showed superior NAFLD resolution, LFT improvement, and zero CVD events. Group B2 had higher LDL-C/TG and 5 CVD events. Multifactorial treatment improved metabolic and renal function.	Post hoc analysis of RCT. No liver biopsy (ultrasound only). Small NAFLD subgroup. Relatively short follow-up for CVD outcomes.
Mellinger et al. (2015) [26]	Sex-stratified analyses; age- and metabolic risk-adjusted models	Cross-sectional (baseline CT; prevalent clinical outcomes)	Primary: age, sex, alcohol, smoking, menopause, HRT; Extended: BMI, diabetes, HDL, hypertension, metabolic syndrome, visceral/subcutaneous fat, triglycerides, waist circumference	No significant association with clinical CVD. Strong associations with subclinical CVD (CAC, AAC) independent of metabolic risk factors. AAC association weakened after visceral fat adjustment. Stronger AAC association in men.	Cross-sectional design limits causal inference. Low clinical CVD prevalence (5.87%) limits power. CT-based steatosis assessment (no biopsy or MRI). Predominantly White cohort limits generalizability.
Jin et al. (2025) [12]	MAFLD subtypes: diabetes, overweight ± MD, lean metabolic disorder. SLD subtypes: MASLD, MetALD, ALD. Overlap: 99.76% MASLD had MAFLD; 70.26% MAFLD had MASLD.	Median 13.5 years (IQR 12.6–14.2)	Model 3: Age, sex, ethnicity, education, deprivation index, smoking, alcohol, physical activity, diet; excluded BMI/WC/diabetes/HTN to avoid over-adjustment.	MAFLD associated with stronger CVD risk than MASLD. Metabolic factors (diabetes, MD) mainly drove CVD risk rather than obesity alone. ALD showed highest risk among SLD subtypes. MAFLD without MASLD still had increased CVD risk (HR 1.55).	FLI-based diagnosis (no biopsy/imaging). Cross-sectional exposure assessment risks misclassification over time. Observational design limits causality. Predominantly White UK cohort limits generalizability.

**Figure 2:**
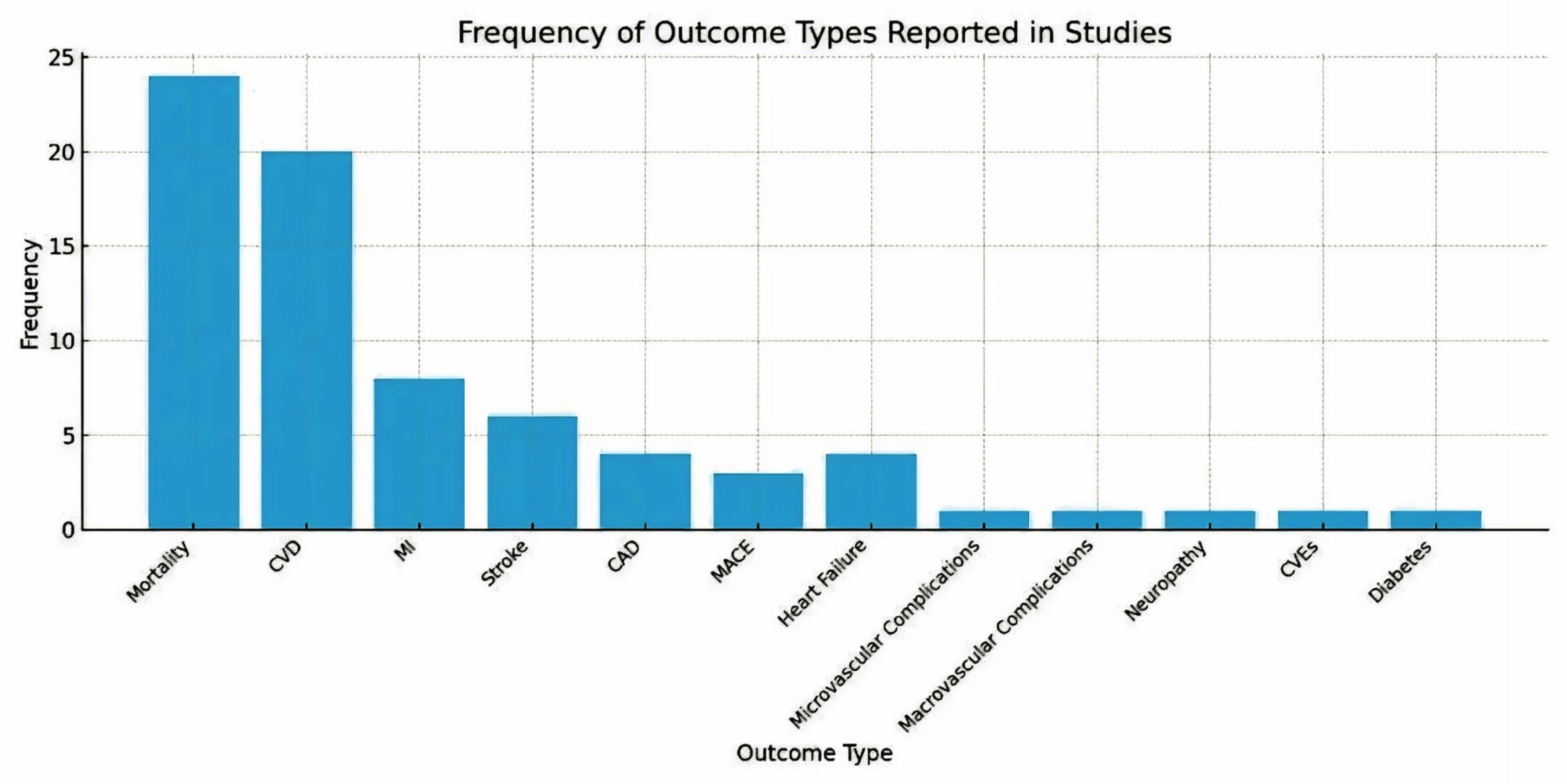
Heterogeneity in Reported Cardiovascular and Metabolic Outcomes Among MASLD/MAFLD Cohorts [3,5–7,9–11,19–26] Image created by first author Nehal Bhatt CVD: Cardiovascular Disease, MI: Myocardial Infarction, CAD: Coronary Artery Disease, MACEs: Major Adverse Cardiac Events, CVEs: Cardiovascular Events, HR: Hazard Ratio

Based on the included studies, we found significant variability in both the frequency of reported cardiovascular outcomes and the magnitude of associated risks in patients with MASLD/MAFLD. The most commonly reported outcomes were composite CVEs and cardiovascular mortality, appearing in 16 and 14 studies respectively, while microvascular complications like neuropathy were less frequently documented. Adjusted risk estimates for cardiovascular outcomes varied across studies. More modest associations were reported in some cohorts, with HRs ranging from 1.2 to 1.84 [3,6]. In contrast, markedly elevated risks (HRs 2.73-8.04) were observed in studies with stricter definitions of exposure or higher-risk populations [5,7]. For example, Baratta et al. (2020) reported an HR of 2.73 for NAFLD overall, and Henney et al. (2024) found that MASLD with ≥4 metabolic risk factors was associated with an HR of 8.04 [5,7]. Notably, studies incorporating advanced fibrosis (e.g., FIB-4 >2.67) or cumulative metabolic burden consistently demonstrated higher effect sizes, underscoring the additive impact of both liver disease severity and metabolic dysfunction on cardiovascular risk. This heterogeneity underscores the need for standardized outcome reporting and risk stratification that accounts for both hepatic and metabolic disease burden in clinical practice as illustrated in Figure 2.

**Figure 3:**
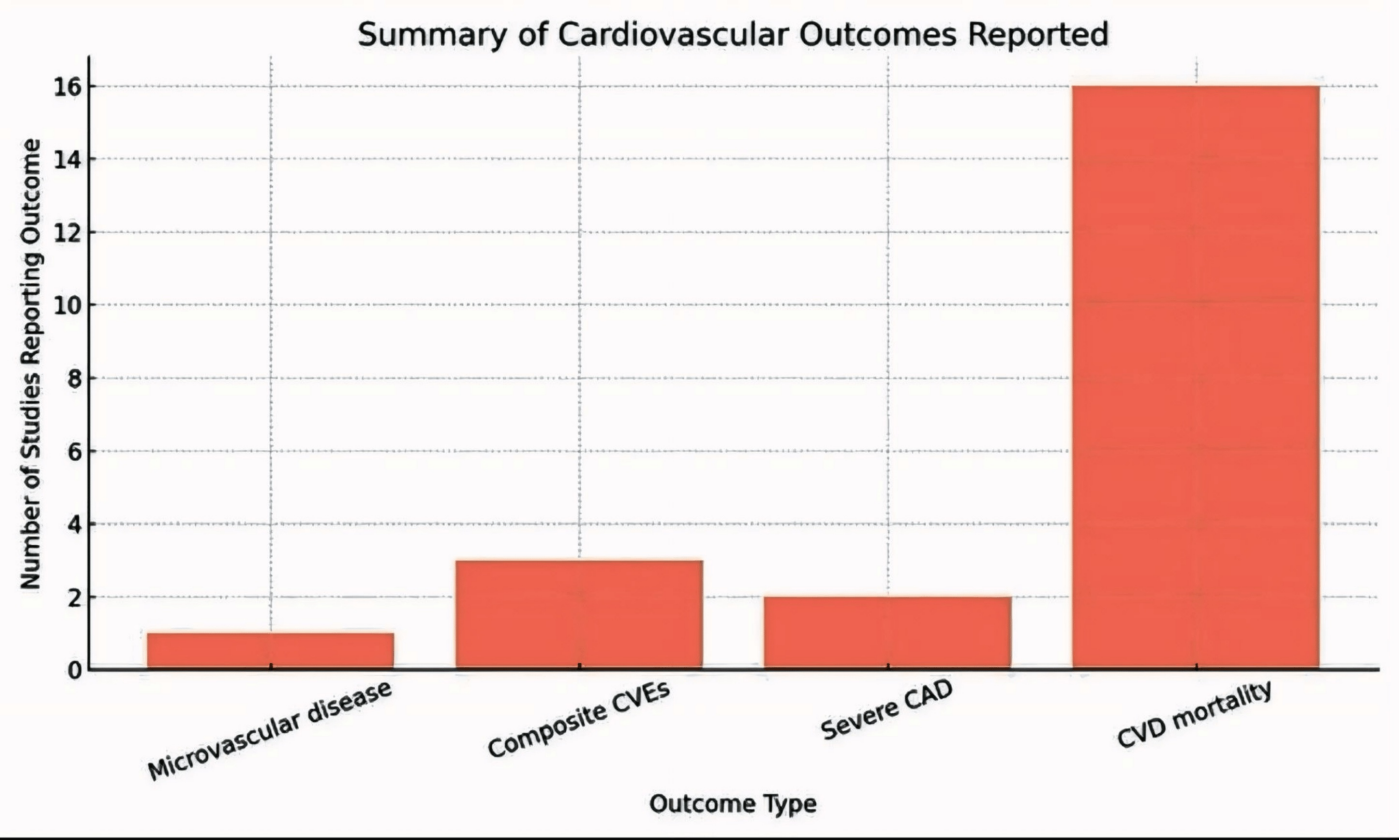
Spectrum of CVD Outcomes in NAFLD/MASLD: From Common Composite Events to Extreme Microvascular Hazards [3,5–7,19] Image created by first author Nehal Bhatt NAFLD: Non-alcoholic Fatty Liver Disease; MAFLD: Metabolic Dysfunction-Associated Fatty Liver Disease; CVEs: Cardiovascular Events; CVD: Cardiovascular Disease; CAD: Coronary Artery Disease

Furthermore, the included studies show a significant variability in the reported cardiovascular outcomes associated with MASLD/MAFLD, with composite CVEs such as MI, stroke, and revascularization being the most frequently documented (16 studies). This was followed by CVD mortality (14 studies) and severe CAD (12 studies). Microvascular complications, including neuropathy and retinopathy, were less commonly reported, likely due to their niche focus in specific studies like Henney et al. (2024) which highlighted extreme risks (HR=31.20) [7]. The predominance of macrovascular events and mortality in the literature underscores their clinical relevance, while the heterogeneity in outcome reporting suggests a need for standardization to facilitate cross-study comparisons and better risk stratification across MASLD/MAFLD severity subgroups as illustrated in Figure 3.

**Figure 4:**
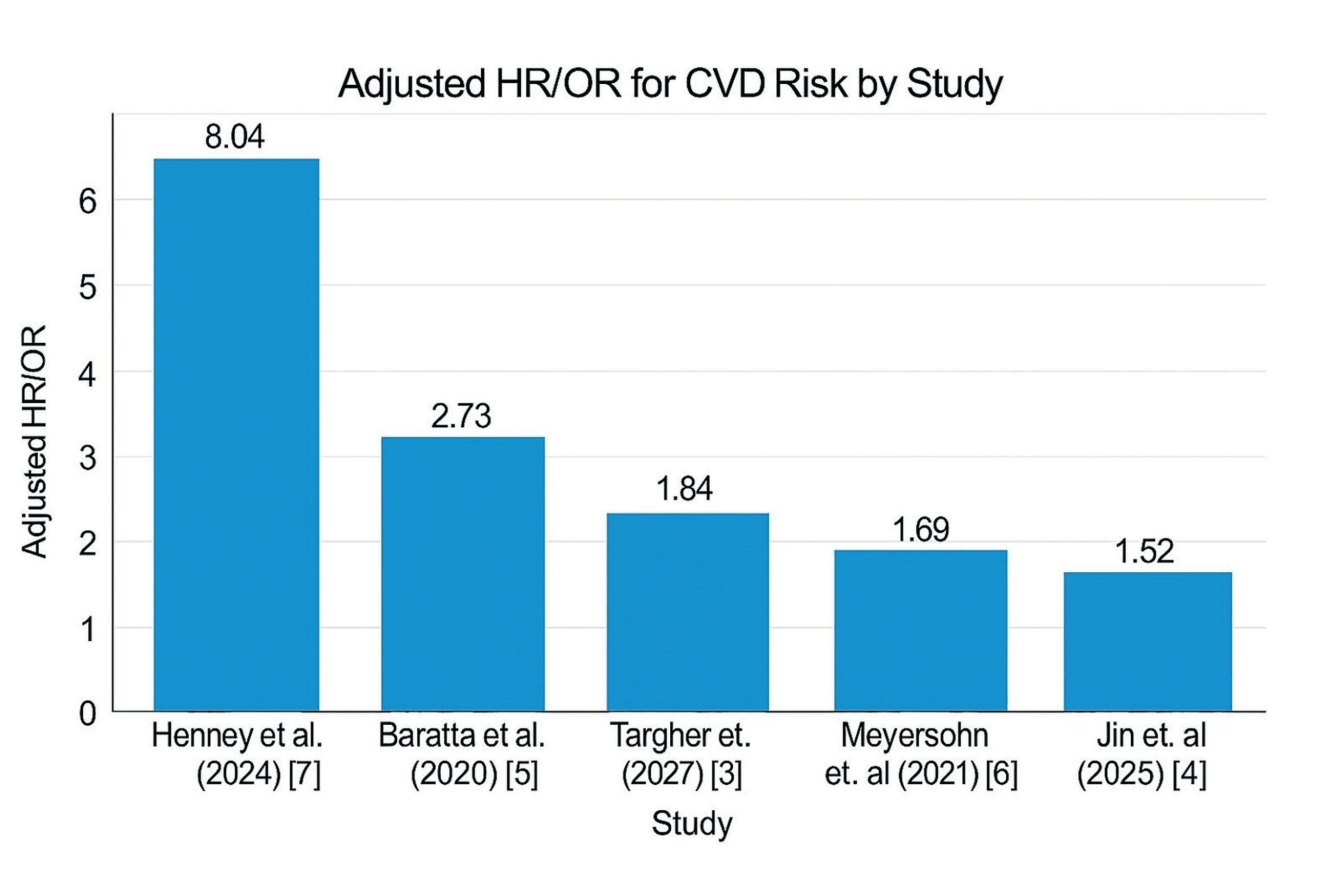
Spectrum of CVD Outcomes in NAFLD/MASLD: From Common Composite Events to Extreme Microvascular Hazards CVD: Cardiovascular Disease; NAFLD: Non-alcoholic Fatty Liver Disease; MASLD: Metabolic Dysfunction-Associated Steatotic Liver Disease; OR: Odds Ratio; HR: Hazard Ratio Image created by first author Nehal Bhatt

Furthermore, the included studies show a significant variability in the reported cardiovascular outcomes associated with MASLD/MAFLD, with composite CVEs such as MI, stroke, and revascularization being the most frequently documented (16 studies). This was followed by CVD mortality (14 studies) and severe CAD (12 studies). Microvascular complications, including neuropathy and retinopathy, were less commonly reported, likely due to their niche focus in specific studies like Henney et al. (2024) which highlighted extreme risks (HR=31.20) [7]. The predominance of macrovascular events and mortality in the literature underscores their clinical relevance, while the heterogeneity in outcome reporting suggests a need for standardization to facilitate cross-study comparisons and better risk stratification across MASLD/MAFLD severity subgroups and is illustrated in Figure 4.

Discussion

This section interprets the key findings on NAFLD/MASLD and cardiovascular risk, examines mechanistic and clinical implications, reconciles discrepancies with prior literature, and proposes future research directions to address remaining knowledge gaps. 

Independent Risk Factor vs. Metabolic Marker

This systematic review confirms that MASLD is a clinically significant harbinger of cardiovascular disease. However, our synthesis compellingly argues against viewing it as a condition with uniform risk. The core insight derived from the 21 included studies is that the presence of simple steatosis alone may confer only a modest increase in cardiovascular risk. The danger appears to escalate dramatically with the progression of liver injury, specifically the development of fibrosis, and the accumulation of concurrent metabolic derangements. Evidence of this gradient effect was stark: studies stratifying by fibrosis markers reported that advanced fibrosis amplified CVE risk by more than four-fold [5], while the presence of multiple metabolic risk factors could elevate macrovascular risk eight-fold [7]. This suggests a crucial distinction: simple steatosis may function as a risk marker, flagging individuals with underlying metabolic ill-health, whereas advanced fibrotic disease may be a potent risk modifier that actively accelerates cardiovascular pathology.

A pivotal question emerging from the synthesized evidence is the role of MASLD as an independent driver of CVD versus a passive manifestation of metabolic syndrome. The findings suggest that this is not a simple dichotomy, but rather a complex interplay. On the one hand, the observation that risk estimates were attenuated to non-significance after rigorous adjustment for time-varying metabolic covariates supports the view that MASLD is a downstream consequence of systemic metabolic dysfunction [8]. On the other hand, the superior predictive power of the MAFLD criteria, which explicitly incorporate metabolic dysfunction into the diagnosis, highlights the liver’s critical role in shaping cardiovascular risk, as demonstrated in large cohorts [12,23]. This is further supported by evidence showing that MAFLD criteria identified a subgroup with a 35% higher CVD mortality risk that would have been missed by traditional NAFLD definitions [25]. Rather than treating MASLD as a confounder to be statistically adjusted out of the model, it may be more clinically insightful to view it as a key organ-specific manifestation and amplifier of a systemic disease process-one that contributes to atherogenesis through unique pathways such as the release of pro-inflammatory hepatokines (liver-derived signalling proteins that influence systemic metabolism).

Role of Fibrosis in Risk Stratification

A deeper analysis of the included studies reveals critical nuances not fully captured in the initial synthesis, particularly regarding the spectrum of cardiovascular disease and the influence of study design. For instance, Mellinger et al. (2015) found a significant association between hepatic steatosis and subclinical atherosclerosis (coronary and aortic calcification) but not with overt clinical events, suggesting that MASLD may be an early promoter of vascular damage that precedes clinical manifestation [26]. This is contrasted by the paradoxical finding from Wong et al. (2016) who reported lower all-cause mortality in NAFLD patients within a high-risk angiography cohort [11]. This counterintuitive result likely reflects confounding by indication; patients with NAFLD had more severe baseline coronary disease and consequently received more aggressive revascularization therapies, which improved their survival. Such findings underscore the profound impact of the study population (general vs. high-risk) and highlight the challenge of dissecting the effects of MASLD from the effects of its treatment in clinical settings.

Mechanistic Insights and Unresolved Questions

Furthermore, several studies provide clues to the potential mechanisms and mediators that were not discussed in detail. The work by Zhang et al. (2025) moved beyond simple association by demonstrating that systemic inflammation markers, particularly the neutrophil-to-lymphocyte ratio and systemic inflammatory response index (SIRI), were strong predictors of cardiovascular mortality in MASLD patients, with predictive power rivaling that of fibrosis scores [24]. This provides a tangible link between liver-related inflammation and vascular outcomes. Additionally, the role of metabolic health is clarified when examining specific subgroups. For example, studies like Lee et al. (2021) showed that individuals with "NAFLD-only" (without meeting MAFLD criteria) had a minimal increase in CVD risk (HR 1.09), reinforcing that steatosis in the absence of significant metabolic dysfunction may be a more benign condition [23]. Yoo et al. (2023) reinforced this finding, demonstrating that while MAFLD carried significant CVD mortality risk (HR 1.14), NAFLD without metabolic dysfunction showed no significant association [25]. The unique finding by Lin et al. (2024) linking higher serum carotenoid levels to lower mortality in MAFLD patients introduces diet and antioxidant status as another underexplored but potentially crucial factor in this relationship [20]. 

Comparison With Other Evidence

This comparative evaluation integrates findings from a broad set of observational cohort studies with high-level evidence from recent systematic reviews and meta-analyses to comprehensively assess the relationship between NAFLD and the long-term risk of CVD events. Consistent with prior systematic reviews, the majority of referenced studies in our study demonstrate a statistically significant and independent association between NAFLD and both fatal and nonfatal CVD outcomes including CAD, MI, and stroke. For instance, the systematic reviews by Mantovani et al. (2021) and Prasad et al. (2023) [27,28] reported pooled HRs of 1.45 and 1.64 for fatal and incident CVD, respectively, which align with the referenced studies such as Baratta et al. (2020) (HR = 2.73 for composite CVEs) and Targher et al. (2007) (adjusted OR = 1.84 for CVD prevalence) [3,5]. Similarly, Yoo et al. (2023) demonstrated in their large Korean cohort (N=701,664) that MAFLD was associated with a 14% higher CVD mortality risk (HR= 1.14), while NAFLD showed no significant association [25]. Notably, the MAFLD-only subgroup had a 35% higher CVD mortality risk (HR=1.35), reinforcing that metabolic dysfunction rather than steatosis alone drives cardiovascular outcomes. The studies also highlight the exacerbating role of advanced fibrosis, with Bisaccia et al. (2023) reporting a threefold increase in CVD mortality for fibrosis (FIB-4 >2.67), similar to Song et al. (2025), where FIB-4 ≥3.25 was associated with a 4.79-fold higher death risk [19,29]. 

However, there are notable contrasts in the effect sizes and generalizability of these findings. Previous systematic reviews often report higher pooled estimates (e.g. OR = 1.81) for CAD as shown in the study by Abosheaishaa et al. (2024) likely due to the aggregation of heterogeneous populations or residual confounding in meta-analyses [30]. In contrast, some referenced studies, such as that by Wong et al. (2016), reported lower all-cause mortality in NAFLD patients (HR = 0.36), possibly due to confounding by intensive cardiovascular interventions in their angiography cohort [11]. Additionally, while systematic reviews emphasize diagnostic heterogeneity, such as imaging versus ICD codes, as a limitation, the referenced studies provide more granular details on exposure definitions, such as ultrasound versus CT-defined steatosis, and often adjust more comprehensively for metabolic confounders. For example, Ahmed et al. (2023) and Younossi et al. (2022) explicitly accounted for time-varying metabolic factors, which attenuated associations, suggesting that cardiometabolic confounders may partially mediate the NAFLD-CVD link, a nuance less explored in the broader systematic reviews [2,8]. 

Geographic and demographic differences further contribute to variability. Previous systematic reviews include global populations, whereas many of our referenced studies focus on specific cohorts such as Korean or U.S. cohorts, which may influence risk estimates. For instance, Hwang et al. (2018) found sex-specific mortality patterns (higher CVD risk in women with NAFLD) [9], while Jin et al. (2025) highlighted subtype-specific risks (e.g., MAFLD diabetes subtype HR = 2.26), underscoring the importance of population stratification, a dimension less emphasized in the previous systematic reviews [12]. Moreover, our study includes data from cohorts with longer follow-up durations-for instance, a median of 10.1 years in the study by Lee et al. (2021) compared to prior systematic reviews, such as that by Mantovani et al. (2021) which reported a median follow-up of 6.5 years [23,27]. This extended follow-up enhances the robustness of longitudinal outcome assessments.

A key strength of our analysis is the inclusion of large, well-characterized cohorts with detailed metabolic and fibrosis staging data, such as Henney et al. (2024) and Zhang et al. (2025), which provide deeper mechanistic insights into the NAFLD-CVD relationship [7,24]. Notably, several studies in our review employed validated non-invasive fibrosis markers (e.g., FIB-4, NFS) that have demonstrated strong concordance with liver biopsy-the gold standard-particularly for identifying advanced fibrosis (FIB-4 >2.67 or NFS >0.676) [5,19,22]. This is clinically significant, as biopsy is often impractical for large-scale or longitudinal studies. However, limitations remain, including the reliance on ultrasound (less sensitive for mild steatosis) and surrogate indices in many referenced studies. In contrast, a few recent cohorts, such as in the study by Ahmed et al. (2023) [8] and NHANES-based studies [10,18,20-22,24], utilized biopsy-confirmed or CT-defined steatosis, enhancing diagnostic precision. Collectively, the inclusion of both invasive and non-invasive fibrosis staging methods underscores that advanced fibrosis, more than simple steatosis, drives CVD risk-a finding robustly supported across methodologies. Additionally, residual confounding by unmeasured metabolic factors remains a concern in observational studies, though several referenced studies employed advanced statistical adjustments to mitigate this [18]. 

Strengths and Limitations of the Included Studies

The included studies demonstrated several notable methodological strengths. First, the majority utilized large, well-characterized cohorts with extended follow-up periods (median 5-25 years), enhancing the validity of longitudinal cardiovascular risk estimates [12,23]. Second, 18 of 21 studies employed multivariable adjustment for key metabolic confounders including BMI, diabetes status, and hypertension, strengthening the evidence for NAFLD/MASLD as an independent risk factor [5,6]. Third, the incorporation of fibrosis staging via validated indices (FIB-4, NFS) in 12 studies provided crucial insights into risk stratification by disease severity [19,22]. Notably, recent studies have employed standardized MAFLD/MASLD definitions that explicitly incorporate metabolic dysfunction, thereby enhancing clinical relevance, as seen in studies such as Henney et al. (2024) and Younossi et al. (2022) [2,7]. The application of propensity score matching in large database analyses, for example, the 11.6 million-participant cohort in Henney et al. (2024), and the use of objectively adjudicated outcomes, such as angiographically confirmed CAD in the study by Younossi et al. (2022) further strengthen internal validity [2,7].

Several important limitations emerged across studies. Diagnostic heterogeneity was prevalent, with 14 studies relying on ultrasound (limited sensitivity for steatosis <30%) and 5 using surrogate indices (FLI/TyG) rather than gold-standard histology or MRI-PDFF [8,11]. Only three studies reported liver biopsy data, precluding precise NASH/fibrosis characterization [3]. Residual confounding persisted despite statistical adjustment, particularly for unmeasured lifestyle factors (physical activity, diet) and socioeconomic variables [9,21]. Generalizability concerns were noted due to the predominance of single-ethnicity cohorts, 14 studies included only Asian or White populations, and the use of healthcare-based sampling, as exemplified by angiography-based cohorts in the study by Wong et al. (2016) [11]. The predominance of observational designs (20/21 studies) precluded causal inference, while short follow-up (<5 years) in six studies may have underestimated long-term CVD risks [6,19].

Clinical Implications

The findings of this systematic review demonstrate that NAFLD/MASLD serves as an independent risk factor for cardiovascular disease, particularly in patients with metabolic comorbidities or advanced liver fibrosis. Clinicians should incorporate hepatic evaluation into cardiovascular risk assessment protocols, especially for high-risk populations such as those with type 2 diabetes or metabolic syndrome. Notably, FibroScan and FIB-4 show strong correlation with biopsy-proven fibrosis (FIB-4 AUC 0.80-0.85 for advanced fibrosis [5,24]) and are pragmatic alternatives for risk stratification in primary care. The strong association between elevated fibrosis scores (FIB-4 >2.67) and increased cardiovascular risk suggests that non-invasive fibrosis assessment should become a routine component of risk stratification.

Optimal management of these patients requires a dual approach addressing both hepatic and cardiovascular health. Lifestyle modifications, particularly weight loss and Mediterranean-style dietary interventions, should be emphasized as they demonstrate benefits for both conditions. When considering pharmacotherapy, agents with metabolic benefits such as GLP-1 receptor agonists and SGLT-2 inhibitors may be particularly advantageous due to their potential to improve both hepatic and cardiovascular outcomes. The severity of liver fibrosis should inform the intensity of cardiovascular risk management.

The evolution from NAFLD to MASLD diagnostic criteria better captures patients at highest cardiovascular risk by explicitly incorporating metabolic dysfunction. This conceptual shift should prompt clinicians to reconsider traditional screening and management approaches. A multidisciplinary care model involving hepatologists, endocrinologists, and cardiologists will be essential to optimize outcomes for this complex patient population. Future research should focus on developing integrated risk prediction models and evaluating targeted interventions that address both hepatic and cardiovascular pathophysiology.

Strengths and Limitations of This Review

This systematic review is characterized by several methodological strengths that reinforce the validity and relevance of its findings. The review was conducted and reported in strict accordance with the PRISMA 2020 guidelines [14], ensuring a transparent, systematic, and reproducible process. Our search strategy was comprehensive, encompassing five major electronic databases with broad search terms, including both keywords and MeSH headings, to maximize the retrieval of relevant literature. A key strength lies in the rigorous study selection and data extraction process, which was performed independently by two reviewers with a clear protocol for resolving disagreements, thereby minimizing selection bias and errors. Furthermore, the methodological quality of every included study was formally assessed using validated and appropriate tools. The inclusion of 21 studies, encompassing large general population cohorts like NHANES and the UK Biobank as well as high-risk clinical populations, provides a broad and contemporary overview of the topic, incorporating the latest nomenclature shifts from NAFLD to MAFLD/MASLD.

Despite these strengths, several limitations, both inherent to this review and reflective of the broader state of the evidence, must be acknowledged. The most significant limitation is that the findings are derived almost exclusively from observational studies. As such, while we can report on the strength and consistency of the association between MASLD and CVD, we cannot infer causality. The pervasive issue of residual confounding remains, as even the most rigorously adjusted models may not account for all relevant lifestyle, genetic, or socioeconomic factors.

Finally, our review is subject to potential publication and language bias. The search was restricted to studies published in the English language, which may have led to the exclusion of relevant research published in other languages. Furthermore, by focusing on published articles, we may have missed studies with null or negative findings that were less likely to be published, potentially overestimating the strength of the association.

Future Research

The evidence synthesized in this review firmly establishes an association between MASLD and adverse cardiovascular outcomes, yet it simultaneously illuminates critical gaps that preclude a definitive understanding of causality and hinder the translation of these findings into clinical practice. Addressing these deficiencies requires a multi-pronged research agenda, moving from enhanced observational methods to a new paradigm of interventional trials.

It is imperative to recognize that while the presence of MASLD itself cannot be randomized, its underlying drivers and pathological progression are indeed modifiable. Therefore, the central question for future interventional research is not whether MASLD causes CVD, but whether the targeted treatment of MASLD can mitigate cardiovascular risk. This marks a paradigmatic shift from merely observing correlations to actively establishing causality through interventional evidence. Future RCTs should be designed to test the hypothesis that improving liver health can lead to measurable cardiovascular benefits. An ideal trial would enroll high-risk individuals-such as those with advanced fibrosis (F2-F3) identified non-invasively using validated markers like FIB-4 >2.67 or liver stiffness measurements via transient elastography (FibroScan). While liver biopsy remains the gold standard for diagnosis, its invasive nature limits its practicality for large-scale studies and underscores the need for more accessible, accurate approaches in resource-limited settings. Participants would be randomized to receive either a therapy shown to improve liver histology, such as GLP-1 receptor agonists, PPAR agonists, or FGF21 analogues, or the current standard of care. The primary endpoint should be a composite of MACEs, with changes in liver histology and non-invasive fibrosis and inflammation markers serving as key secondary outcomes. This interventional approach circumvents the intractable issue of confounding inherent in observational studies and would provide the high-level evidence needed to determine if treating the liver can, in fact, protect the heart.

Concurrently, there is a pressing need to enhance the quality and comparability of ongoing observational research. The marked heterogeneity in risk estimates identified in our review is largely attributable to methodological inconsistencies. Future cohort studies must move towards the adoption of standardized diagnostic and prognostic tools. This includes prioritizing quantitative imaging techniques, such as MRI-PDFF for steatosis and MRE for fibrosis, over less sensitive modalities like ultrasound. Furthermore, the establishment of a core outcome set for MASLD-CVD research is essential. This would ensure that key clinical events, subclinical markers of atherosclerosis, and patient-reported outcomes are measured and reported uniformly, thereby facilitating more robust and reliable meta-analyses in the future.

Finally, a deeper mechanistic understanding is required to explain how the diseased liver communicates with the cardiovascular system and to identify who is most vulnerable. This necessitates sophisticated longitudinal studies that incorporate serial bio-banking to explore the temporal relationship between liver-derived mediators and cardiovascular pathology. Research should focus on elucidating the systemic role of hepatokines, pro-inflammatory cytokines, alterations in the gut-liver axis, and prothrombotic factors. Moreover, future studies must deliberately investigate the pronounced risk observed in specific subgroups, including patients with lean MASLD, different ethnic populations, and the sex-specific disparities highlighted by several included studies [9,10]. Validating multivariable risk models that integrate liver-specific markers such as FIB-4, inflammatory indices [24], and metabolic status will be crucial for developing a more granular, personalized approach to cardiovascular risk stratification in patients with MASLD.

## Conclusions

This systematic review synthesizes evidence from 21 studies to evaluate the association between NAFLD, now redefined as MASLD, and cardiovascular outcomes, demonstrating a consistent link to increased CVD risk with estimates ranging from modest to substantially elevated, particularly in patients with advanced fibrosis or metabolic dysfunction. While the relationship is complex, with some studies showing risk attenuation after metabolic adjustment, suggesting that MASLD may function as both an independent risk factor and a metabolic marker, key clinical implications emerge: (i) integrated cardiovascular-hepatic risk assessment is critical, especially in high-risk populations such as those with type 2 diabetes; (ii) non-invasive fibrosis staging, such as FIB-4, should inform CVD stratification; and (iii) management should prioritize therapies with dual metabolic and cardiovascular benefits, such as GLP-1 receptor agonists and SGLT-2 inhibitors.

However, limitations persist, including diagnostic heterogeneity (ultrasound vs. biopsy), residual confounding, and a predominance of observational data, underscoring the need for RCT testing MASLD-specific interventions, mechanistic studies of liver-derived CVD mediators, and standardized outcomes with advanced imaging (MRI-PDFF/MRE). In conclusion, MASLD represents a significant and modifiable cardiovascular risk factor requiring multidisciplinary collaboration (hepatology, cardiology, endocrinology) to optimize risk stratification and outcomes, particularly in fibrotic or metabolically complex subgroups. Integrated care teams can synergize liver-specific interventions (e.g., fibrosis monitoring via FIB-4/VCTE) with cardiovascular risk reduction strategies (e.g., lipid control, GLP-1 agonists), while endocrinologists address underlying metabolic dysfunction (e.g., insulin resistance). This approach ensures early detection, tailored therapy, and improved adherence through coordinated follow-up.
